# Utilizing a Simple Method for Stoichiometric Protein Labeling to Quantify Antibody Blockade

**DOI:** 10.1038/s41598-019-43469-z

**Published:** 2019-05-07

**Authors:** Rachel Friedman Ohana, Robin Hurst, Mike Rosenblatt, Sergiy Levin, Thomas Machleidt, Thomas A. Kirkland, Lance P. Encell, Matthew B. Robers, Keith V. Wood

**Affiliations:** 1 Promega Corporation, 2800 Woods Hollow Rd, Madison, WI 53711 USA; 2 Promega Biosciences LLC, 277 Granada Dr, San Luis Obispo, CA 93401 USA

**Keywords:** Chemical modification, Antibody therapy

## Abstract

Ligand binding assays routinely employ fluorescently-labeled protein ligands to quantify the extent of binding. These ligands are commonly generated through chemical modification of accessible lysine residues, which often results in heterogeneous populations exhibiting variable binding properties. This could be remedied by quantitative, site-specific labeling. Recently, we reported on a single-step method integrating recombinant protein purification with 2-cyanobenzothiazole (CBT) condensation for labeling a proteolytically exposed N-terminal cysteine. Here, using three growth factors, we show that unlike random lysine labeling, this site-specific approach yielded homogeneous populations of growth factors that were quantitatively labeled at their N-termini and retained their binding characteristics. We demonstrate the utility of this labeling method through the development of a novel assay that quantifies the capacity of antibodies to block receptor-ligand interactions (i.e. antibody blockade). The assay uses bioluminescence resonance energy transfer (BRET) to detect binding of CBT-labeled growth factors to their cognate receptors genetically fused to NanoLuc luciferase. The ability of antibodies to block these interactions is quantified through decrease in BRET. Using several antibodies, we show that the assay provides reliable quantification of antibody blockade in a cellular context. As demonstrated here, this simple method for generating uniformly-labeled proteins has potential to promote more accurate and robust ligand binding assays.

## Introduction

Ligand binding assays are routinely used to measure interactions of protein ligands with cellular receptors, antibodies and other macromolecules^1–4^. The quality of these assays relies on their capacity to represent native biology^1^. Accordingly, when fluorescently-labeled protein ligands are employed to facilitate detection and quantification of binding to a target, it is important that the labeling of these ligands does not significantly alter their binding properties^1,2,4,5^. Additionally, the ability to reproducibly generate well-characterized, fluorescently-labeled protein ligands is essential to assay robustness.

Fluorescently-labeled protein ligands can be generated by a variety of methods^6–11^. One of the most common is random chemical modification of accessible lysine residues and N-termini by N-hydroxysuccinimidyl (NHS) esters^6,7,11^. The popularity of this approach is likely due to its ease of use and the fact that labeling reagents are commercially available. However, since lysines are abundant on protein surfaces^10^ and are frequently involved in binding interactions, exhaustive labeling could be disruptive to protein interactions and function. Consequently, reaction conditions are routinely adjusted so that only a subset of lysines are modified. This inevitably results in heterogeneous populations of labeled proteins, which often exhibit variable binding properties and biological potencies^6,7,11^. Labeling proteins with more than one fluorophore can also decrease protein solubility and reduce fluorescence intensity due to proximity quenching^6^.

The ability to quantitatively label a protein with a single fluorophore at a specific site would eliminate population heterogeneity and reduce the risk of altering a ligand’s binding properties. Yet, despite the plethora of reported site-specific labeling techniques^7–10^, finding a method that is robust, simple and achieves stoichiometric labeling (i.e. one fluorescent label per protein) is not trivial. For example, enzymatic approaches utilizing peptide ligases are highly specific but can suffer from inefficiency^8,10^. Other approaches that rely on genetic incorporation of unnatural amino acids bearing biorthogonal functional groups for subsequent labeling can provide specificity but are prone to protein truncation and inefficient incorporation^8–10^. Overall, the wide use of such site-specific labeling methods has been limited either by their complexity, labeling efficiency, or both.

**Figure 1:**
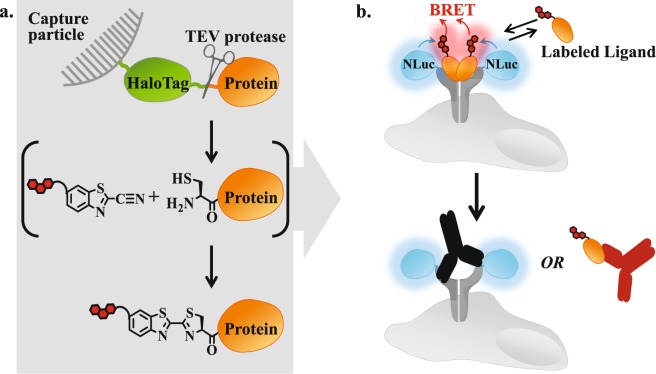
Generation of labeled protein ligands for quantification of antibody blockade by BRET. (**a**) Illustration of a single-step method integrating HaloTag-based recombinant protein purification with CBT condensation for stochiometric labeling of an N-terminal cysteine that is proteolytically exposed during purification. (**b**) Illustration of a BRET assay that quantifies antibody blockade on the surface of living cells. Equilibrium binding of a fluorescently-labeled protein ligand to its cognate receptor that is genetically fused to NanoLuc results in BRET. The capacity of antibodies that recognize either the ligand or the receptor to physically block this interaction is quantified through a decrease in BRET.

Recently, we described a single-step method that integrates HaloTag-based recombinant protein purification^12–14^ with 2-cyanobenzothiazole (CBT) condensation^15,16^ for efficient labeling of an N-terminal cysteine that is proteolytically exposed during purification (Fig. 1a)^17^. This bioorthogonal condensation offers a high degree of selectivity that relies on the distinctive reactivity of CBT toward 1, 2-aminothiols. While 1, 2-aminothiols are not natively present in proteins, they can be introduced by appending an N-terminal cysteine. Using three growth factors (epidermal growth factor (EGF)^18^, vascular endothelial growth factor (VEGF_165_a)^19^ and platelet-derived growth factor (PDGF-BB)^20^) as model systems, we compared this straightforward site-specific CBT-labeling method to the common and facile random modification of lysine residues. Unlike random labeling, the CBT method reproducibly yielded homogeneous populations of fluorescently-labeled growth factors, which exhibited binding characteristics and bioactivities (i.e. capacities to induce downstream signaling) that were not significantly different from those of their unlabeled counterparts (as determined by one-way ANOVA analysis P > 0.05).

Here, we set out to demonstrate the value of this site-specific CBT-labeling method through the development of a novel assay that quantifies the capacity of antibodies to block receptor-ligand interactions. Such analysis is often required during development of therapeutic antibodies, designed to recognize either cell surface receptors or their corresponding protein ligands, in order to disrupt their interactions and consequently negate aberrant downstream signaling^21,22^. As depicted in Fig. 1b, the assay utilizes BRET^23^ to monitor the interaction of a fluorescently-labeled protein ligand with its cognate receptor that is genetically fused to NanoLuc luciferase^24^. Blockade of this interaction by an antibody that recognizes the receptor, or the ligand is quantified through a decrease in BRET. To test the effectiveness of this BRET-based assay, we utilized the interactions between the three growth factors (EGF, PDGF-BB and VEGF) and their cognate receptors, which have long been targets for cancer therapy^25,26^. Employing several therapeutic and research antibodies we demonstrated the capacity of this assay to reliably quantify blockade efficacies for antibodies that target the receptors or their ligands.

The inherent distance constraints of BRET^23^ permit measurement of molecular proximity in the context of live cells, where cell surface receptors are presented in their native conformation^4,17,27^. In combination with the benefits of site-specific CBT-labeling, this assay can reliably represent endogenous receptors and ligands that the antibodies are intended to recognize and interactions they meant to disrupt. We anticipate that this robust, simple, homogenous assay quantifying physical antibody blockade will complement the suite of functional^25,26^ and biochemical antibody binding assays^21,22^ that are already being used throughout the development of therapeutic antibodies.

## Results

### Generation of fluorescently-labeled growth factors

**Figure 2:**
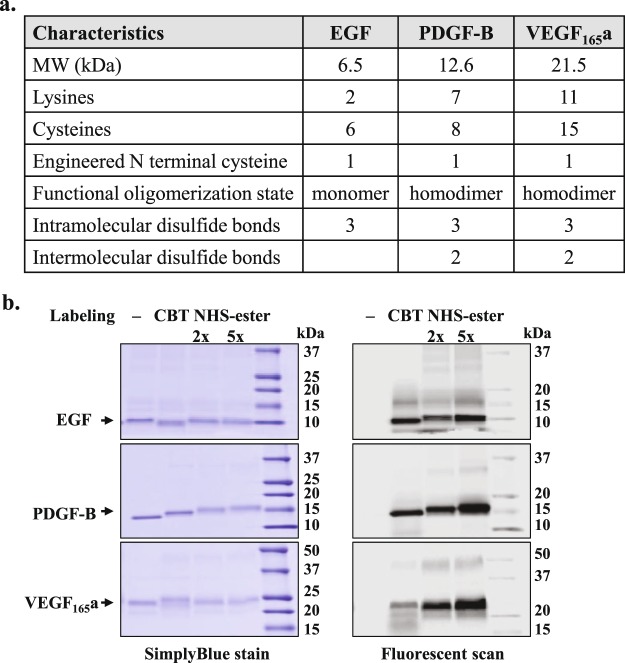
Generation of fluorescently labeled growth factors. (**a**) Properties of growth factors used in the study. (**b**) SDS-PAGE analysis of purified labeled and unlabeled growth factors. Equal amounts of purified proteins were resolved on SDS-PAGE, scanned on a fluorescent imager (Typhoon FLA9500; GE Healthcare using the Cy3 setting) to monitor fluorescent intensity and then stained by SimplyBlue. Images of SimplyBlue stained gels were acquired on ImageQuant LAS4010 (GE Healthcare). Uncropped images are shown in Supplementary Fig. 5.

The robustness of quantitative binding assays that utilize fluorescently-labeled protein ligands stems from the capacity to reproducibly generate those ligands and characterize their binding properties. In this regard, we compared our site-specific CBT-based method with the common random modification by NHS-esters for labeling efficiency, homogeneity, and influence on binding characteristics and bioactivity. To minimize bias, we applied the labeling methods to three growth factors (EGF, PDGF-B and the isoform VEGF_165_a) that varied in size (6.5–21.5 kDa), number of lysines (2–11), number of cysteines (6–15) and functional oligomerization state (Fig. 2a). We chose red emitting Dyomics 605 dye (DY605) as the labeling fluorophore (E_ex_ = 600 nm; E_em_ = 624 nm, in ethanol), because it is a suitable energy acceptor for a NanoLuc energy donor^4,28^ and its hydrophilic nature reduces the possibility of protein precipitation that may result from a high degree of labeling^29^.

Briefly, for the purification of labeled and unlabeled proteins, the growth factors were expressed as secreted N-terminal HaloTag fusions carrying a modified TEV recognition site (EDLYFQC) within the linker separating HaloTag and the growth factors. Subsequent HaloTag-based protein purification^13^ yielded through proteolytic release growth factors having a three-amino acid (CDN) N-terminal appendage. As the degree of random labeling often requires optimization, NHS-ester labeling of the purified growth factors was carried out using a 2 and 5-fold molar excess of DY605-NHS-ester over protein. Generation of CBT-labeled growth factors was performed according to our published single-step purification and labeling method^17^, where the proteolytic release was carried out in the presence of PEG-linked DY605 and CBT conjugate (DY605-PEG-CBT). In addition, since PDFG-B and VEGF_165_a form di-sulfide linked functional homodimers^19,20^, we further confirmed by SDS-PAGE analysis in the presence and absence of 100 mM dithiothreitol (DTT) that these purified growth factors were predominantly present as homodimers (see Supplementary Fig. S1).

To compare the labeling efficiency by the two methods, equal amounts of purified proteins were resolved on SDS-PAGE, scanned on a fluorescent imager to monitor fluorescent intensity and then stained by SimplyBlue to confirm that equal amounts of proteins were loaded onto the gel (Fig. 2b). Generally, higher fluorescence intensity was displayed by the NHS-ester-labeled growth factors, which is likely due to conjugation of multiple fluorophores per protein. Furthermore, the extent of increased fluorescence was correlated to the molar excess of DY605-NHS-ester used in the labeling reactions.

### Assessment of labeling heterogeneity

**Figure 3:**
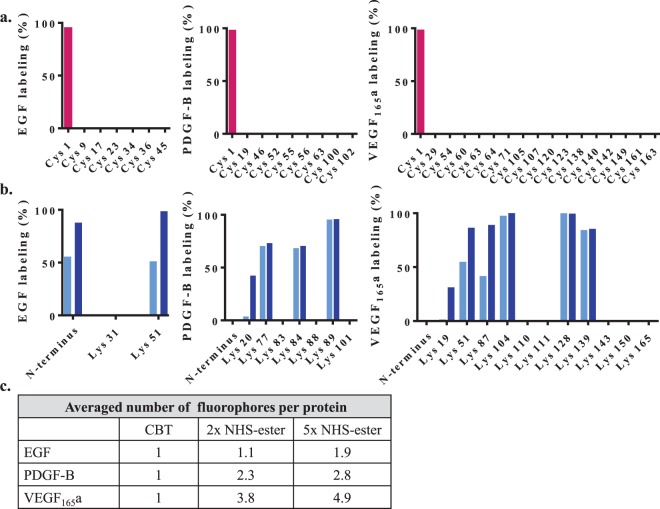
LC-MS/MS profile of CBT and NHS-ester-labeled growth factors. Analysis of growth factors labeled by (**a**) CBT () and (**b**) 2-fold () and 5-fold () molar excess of NHS-ester for the number of modified residues and the extent to which these residues were labeled. Modified residues were identified through increased mass of 1434.42 Da or 973.23 Da for a DY605-PEG-CBT or DY605-NHS-ester modification, respectively. For each modified residue, the degree of labeling was estimated by assessing the fraction that remained unmodified and subtracting it from a theoretical maximum of 100% labeling. To this end, equal amounts of labeled and unlabeled samples were compared for the relative abundance of unmodified peptides encompassing those residues. Relative abundances were derived from the ratio between the integrated peak areas in the Extracted Ion Chromatograms of those unmodified peptides, where the integrated peak area from unlabeled samples represented 100% peptide abundance. The relative abundances of those unmodified peptides in the labeled samples corresponds to the fractions of N-terminal cysteines and lysines that were not modified (see Supplementary Tables S1 and S2). (**c**) Estimated average number of conjugated fluorophores per protein.

To identify residues modified by either DY605-PEG-CBT or DY605-NHS-ester, and to estimate the extent of labeling, we used a liquid chromatography-tandem mass spectrometry (LC-MS/MS) approach (Fig. 3). Briefly, equal amounts of labeled and unlabeled growth factors were digested with multiple proteases to maximize protein coverage and subjected to LC-MS/MS analysis (see Supplementary Fig. S2). DY605-PEG-CBT modification (1434.42 Da) was identified exclusively on the N-terminal cysteine of all three growth factors. None of the other cysteine of EGF, PDGF-B and VEGF_165_a were modified. In contrast, the DY605-NHS-ester modification (973.23 Da) was not only identified on multiple lysines but also on the N-terminus of EGF.

Next, we estimated the degree of labeling for each modified residue by assessing the fraction that remained unmodified and subtracting it from a theoretical maximum of 100% labeling. To this end, we compared equal amounts of labeled and unlabeled samples for the relative abundance of unmodified peptides encompassing those residues (i.e. N-terminal cysteine and lysines shown to be modified). The relative abundance (%) of those peptides in the labeled samples corresponded to the fractions of N-terminal cysteines and lysines that were not modified (see Supplementary Tables S1 and S2).

Using this LC-MS/MS approach, we found that the CBT method yielded selective and near quantitative labeling of the N-terminal cysteine at ≥95% efficiency (Fig. 3a). On the other hand, the random NHS-ester approach resulted in heterogeneous labeling of multiple residues to varying degrees (Fig. 3b). Moreover, the number of conjugated fluorophores varied between 0–2, 0–4 and 0–6 for EGF, PDGF-B and VEGF_165_a, respectively. Considering the extent of labeling of each residue, we estimated the average number of conjugated fluorophores per protein to be in the range of 1.1–1.9, 2.3–2.8 and 3.8–4.9 for EGF, PDGF-B and VEGF_165_a, respectively. These values were dependent on the molar excess of DY605-NHS-ester that was used in the labeling reactions (Fig. 3c). Some lysine residues and N termini were more prone to modification than others. This is probably due to differential accessibility and reactivity.

### Influence of labeling on bioactivity

We further interrogated the influence of the two labeling methods on the bioactivity of these growth factors, which is driven by productive binding to their cognate receptors and subsequent initiation of various signaling pathways^18–20^. To this end, we compared the capacity of labeled and unlabeled growth factors to induce downstream signaling through the expression of a luciferase reporter under the control of relevant transcription factors. We used nuclear factor of activated T cells (NFAT) reporter assay to monitor signaling triggered by binding of aVEGF_165_a homodimer to VEGR2^19,30^, and serum response element (SRE) reporter assay to monitor signaling induced by binding of EGF and PDGF-BB homodimer to EGFR and PDGFRβ, respectively^18,20,31^.

**Figure 4:**
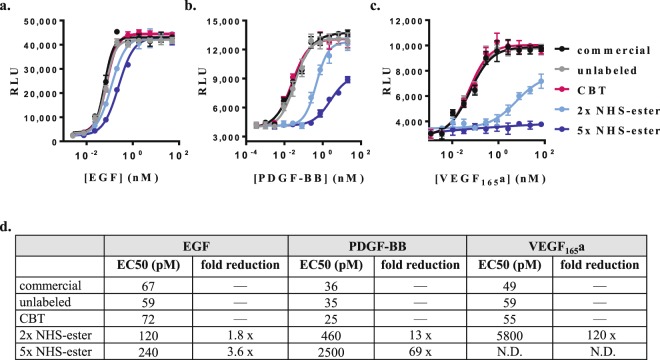
Influence of labeling on growth factors bioactivity. The capacities of labeled and unlabeled growth factors to induce downstream signaling were measured by their ability to stimulate expression of a luciferase reporter (SRE-Re-Luc2P or NFAT-RE-Luc2P) in HEK293 cells that are stably expressing the reporter and transiently expressing a relevant NanoLuc:RTK fusion (n = 3). Expression of SRE-luciferase reporter induced by 6 h stimulation with (**a**) EGF or (**b**) PDGF-BB homodimer and (**c**) expression of NFAT-luciferase reporter induced by 6 h stimulation with VEGF_165_a homodimer. The analysis included growth factors labeled by CBT () or 2-fold () and 5-fold () molar excess of NHS-ester as well as unlabeled growth factors that were purified in the same manner () or obtained from commercial sources (•). (**d**) Summary of observed potencies for labeled and unlabeled growth factors and the influence of labeling method on those potencies.

For each model, we found that unlabeled, CBT-labeled and commercially-obtained growth factors produced almost identical dose dependent cell signaling responses with nearly equal EC50 values (Fig. 4). These results indicated that neither the purification method nor the three-amino acid N-terminal appendage (CDN) or the site-specific CBT labeling had any significant impact on the bioactivity of these growth factors. In contrast, NHS-ester-labeled growth factors evoked substantially weaker cell signaling responses. EGF, PDGF-BB, and VEGF_165_a labeled with 2 and 5-fold molar excess of DY605-NHS-ester induced responses with EC50 values that were 1.8–3.6, 13–39 and ≥120-fold lower than those induced by their unlabeled counterparts, respectively. These results suggest that random NHS-ester labeling can drastically decrease bioactivity in a manner that is dependent on the degree of labeling. It is worth considering that random labeling of lysines, which are possibly involved to variable extents in binding interactions with the cognate receptors, may result in a mixture of subpopulations exhibiting a range of interferences with those interactions and consequently a range of binding affinities and bioactivities. Therefore, it is unclear whether the residual bioactivities are driven by all or just few of the ligand subpopulations that are minimally modified or not modified at all.

### Influence of labeling on binding affinity

**Figure 5:**
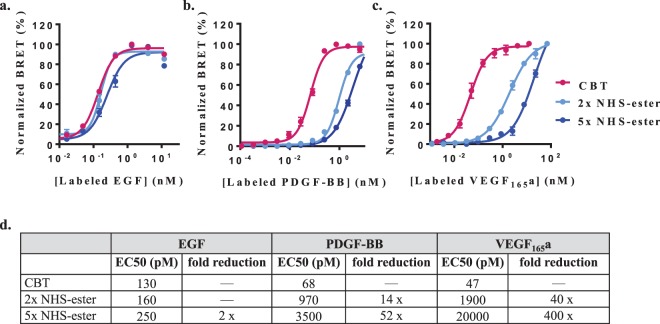
Influence of labeling on growth factors binding to their cognate receptors. Saturation binding of increasing concentrations of (**a**) EGF, (**b**) PDGF-BB homodimer and (**c**) VEGF_165_a homodimer labeled by CBT () or 2-fold () and 5-fold () molar excess of NHS-ester. Binding to the cognate receptors that are genetically fused to NanoLuc in the presence and absence of excess unlabeled equivalents was monitored by BRET (n = 3). Data is expressed as normalized corrected BRET ratios. (**d**) Summary of apparent binding affinities (EC50) for labeled growth factors and influence of labeling methods on EC50 values.

As reporter assays monitor the outcome of signaling cascades, we took a closer look at the influence of the two labeling methods on the initial binding events triggering these downstream pathways. We previously demonstrated that BRET can reveal binding interactions between fluorescently-labeled protein ligands and their cognate receptors that are genetically fused to NanoLuc luciferase^17,27^. Saturation binding experiments using titrated concentrations of labeled growth factors confirmed specific, dose dependent binding to the cognate receptors (Fig. 5). In accordance with the reporter assays, apparent binding affinities of CBT-labeled growth factors were higher than those of their NHS-ester-labeled counterparts. The correlation between estimated degrees of NHS-ester labeling and decreased binding affinities suggests that a high degree of labeling can substantially disrupt binding interactions with the cognate receptors. This is presumably due to increased labeling of lysines that are involved with those interactions. Still, given the heterogeneous nature of NHS-ester labeling, it is unclear if the decreased binding affinities are driven by all or just few subpopulations that are minimally modified.

We further determined binding constants of the labeled and unlabeled growth factors for their cognate receptors (see Supplementary Fig. S3). Equilibrium binding constants (K_D_) of labeled growth factors were derived from saturation binding experiments. IC50 values derived from competitive displacements of labeled growth factors by their unlabeled counterparts were used to calculate binding constants (K_I_) of unlabeled growth factors according to the Cheng-Prusoff equation^32^. This analysis revealed equivalent binding constants for unmodified growth factors (K_I_’s) and CBT-labeled counterparts (K_D_’s), which were in general agreement with reported values^18,33,34^. Further, these results demonstrate for these models the minimally interfering nature of the CBT labeling method.

Notably, similar IC50 and K_I_ values for unlabeled growth factors were derived from competitive displacements of fixed EC80 concentrations of either CBT or NHS-ester-labeled counterparts (see Supplementary Fig. S3). However, the EC80 concentrations of NHS-ester-labeled growth factors used in these experiments, were significantly higher than those of their CBT-labeled counterparts (0–2, 14–50 and 40–400-fold higher for EGF, PDGF-BB and VEGF_165_a labeled with 2 and 5-fold molar excess of DY605-NHS-ester, respectively). These results suggest that mixtures of ligand subpopulations with varying degrees of NHS-ester labeling and subsequently different binding properties, can be used in displacement experiments to derive binding properties of unlabeled ligands. Still, given the decreased binding affinity of these heterogeneous ligand populations, such displacement experiments often require significant quantities of NHS-ester-labeled protein ligands, which may not only be difficult to obtain, but could also reduce assays sensitivity.

### Reproducibility of ligand labeling

**Figure 6:**
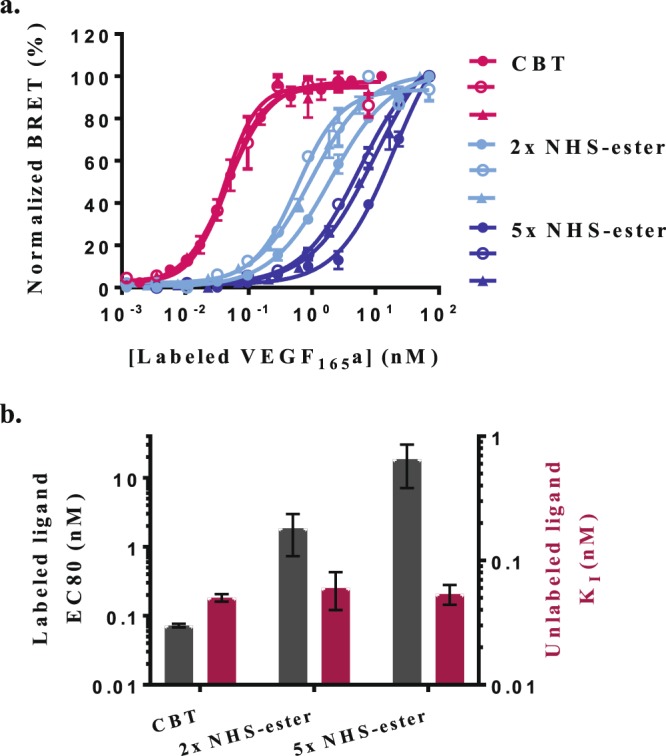
Batch-to-batch variability of VEGF_165_a labeled by CBT and NHS-ester. (**a**) Saturation binding of increasing concentrations of three batches of VEGF_165_a homodimers labeled by CBT () or 2-fold () and 5-fold () molar excess of NHS-ester. Binding to NanoLuc:VEGFR2 fusion in the presence and absence of excess unlabeled VEGF_165_a was monitored by BRET (n = 3). Data is expressed as normalized corrected BRET ratios. (**b**) Summery of EC80 concentrations of labeled growth factors that were used in displacement experiments (black bars) to derive IC50 values and to calculate binding constants (K_I_) for unlabeled growth factors according to the Cheng-Prusoff equation (red bars).

Finally, we compared the two labeling methods for reproducibility. To this end, we generated additional batches of CBT and NHS-ester-labeled VEGF_165_a and tested them for binding affinity to a VEGFR2-NanoLuc fusion (Fig. 6a). Comparison of three independently generated batches of CBT-labeled VEGF_165_a revealed highly reproducible EC50 values (0.045 ± 0.003 nM) with a CV of 6.4%. By contrast, similar analysis for three batches of VEGF_165_a labeled with 2 and 5-fold molar excess of DY605-NHS-ester indicated highly variable EC50 values (1.2 ± 0.7 nM and 12 ± 7 nM, respectively) with CV’s of 60% and 62%, respectively.

Again, similar binding constants (K_I_) for unlabeled VEGF_165_a could be derived from competitive displacements of fixed EC80 concentrations of either CBT or NHS-ester-labeled counterparts. Those EC80 concentrations were remarkably reproducible across three batches of CBT-labeled VEGF_165_a. By contrast, EC80 concentrations for the NHS-labeled counterparts were not only significantly higher but also varied substantially from batch to batch (Fig. 6b). Given this generally low reproducibility, it is likely that EC80 concentrations would need to be determined for each batch of NHS-ester labeled protein ligand.

### Applying CBT-labeled ligands to an assay quantifying antibody blockade

The data presented thus far demonstrates that our CBT-labeling method exhibits several desired features including reproducible and homogeneous ligand labeling with minimal influence on ligand binding properties and bioactivity. These features should be important to the development of robust and quantitative binding assays. We opted to demonstrate this through the development of a quantitative BRET-based assay that measures the capacity of antibodies to block receptor-ligand interactions. The principal assay design is portrayed in Fig. 1b.

**Figure 7:**
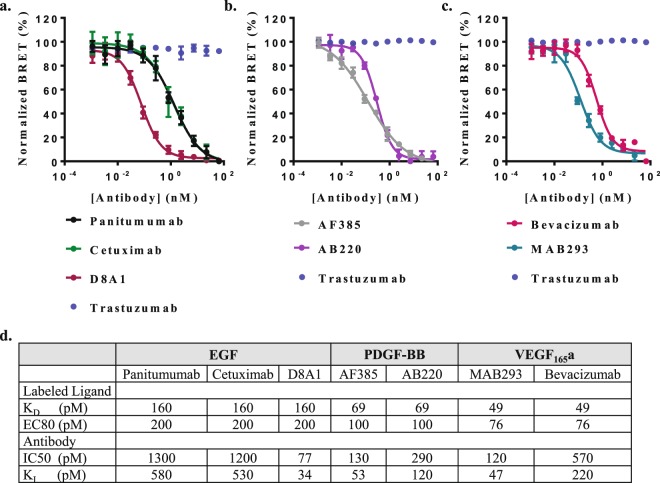
Quantification of antibody blockades. BRET-based quantification of antibodies capacity to block interactions between (**a**) EGF and EGFR, (**b**) PDGF-BB and PDGFRβ as well as (**c**) VEGF_165_a and VGFR2. Cells transiently expressing the relevant NanoLuc:RTK fusion were treated simultaneously with fixed concentrations (EC80) of CBT-labeled growth factors and increasing concentrations of antibodies that recognize either the receptors or the ligands as well as with an irrelevant antibody, Trastuzumab. Data expressed as normalized BRET ratios (n = 4). (**d**) Blockade constants were calculated for each antibody from observed IC50 values according to the Cheng-Prusoff equation.

We used this assay to quantify blockade efficacy of numerous therapeutics and research antibodies that recognize either the receptors or the ligands. The therapeutic antibodies Cetuximab (ERBITUX)^35,36^ and Panitumumab (VECTIBIX)^35,36^, which recognize EGFR, and a research antibody D8A1 recognizing EGF, were tested for their capacity to block the interaction between EGF and EGFR (Fig. 7a). Two research antibodies AF385 and AB220 recognizing PDGFRβ and PDGF-BB, respectively, were tested for their ability to block the interaction of PDGF-BB with PDGFRβ (Fig. 7b). Lastly, two antibodies that recognize VEGF-A, MAB293 and Bevacizumab (Avastin)^34,36^, were evaluated for their ability to block the interaction between VEGF_165_a and VGFR2 (Fig. 7c). To test for assay specificity, we included a non-relevant therapeutic antibody Trastuzumab (HERCEPTIN)^36^, which recognizes HER2. Antibody blockade experiments were performed using increasing concentrations of antibodies in the presence of fixed EC80 concentrations of CBT-labeled growth factors. These experiments confirmed specific, dose-dependent blockade of receptor-ligand interactions by relevant antibodies that recognize either the receptors or the ligands. Furthermore, assay specificity was demonstrated through the failure of an irrelevant antibody, Trastuzumab, to elicit any response. Blockade constants calculated using the Cheng-Prusoff equation^32^ revealed for all tested antibodies sub-nanomolar blockade efficacies, which were in general agreement with reported values^34,36–38^ (Fig. 7d).

Next, we tested how NHS-ester labeling would affect the outcome of this assay format. Using fixed EC80 concentrations of either CBT or NHS-ester-labeled growth factors we found no difference in measurements of blockade efficacies for antibodies that recognize the receptors (see Supplementary Fig. S4). Nonetheless, the generally low affinity and batch to batch variability of NHS-ester labeled ligands presents a significant challenge for their use in the development of robust binding assays.

**Figure 8:**
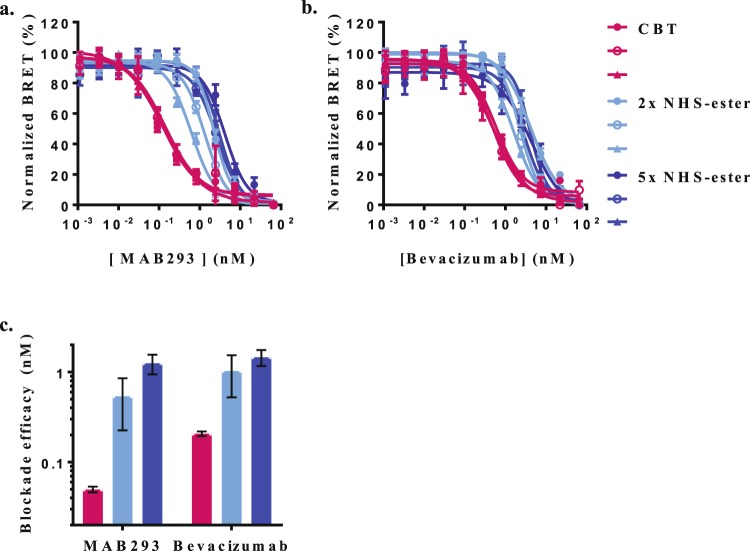
Influence of labeling on blockade efficacies of antibodies that recognize VEGF_165_a. BRET assays quantifying the capacity of (**a**) MAB293 and (**b**) Bevacizumab to block the interactions between a VEGF_165_a homodimer and VGFR2. Cells transiently expressing NanoLuc:VEGFR2 fusion were treated simultaneously with fixed concentrations (EC80) of three batches of VEGF_165_a labeled by CBT () or 2-fold () and 5-fold () molar excess of NHS-ester and increasing concentrations of antibodies. Data expressed as normalized BRET ratios (n = 4). (**c**) Blockade constants were calculated from observed IC50 values according to the Cheng-Prusoff equation. Data is presented as average (K_I_) for three independent batches of VEGF_165_a labeled by CBT (red bars) or 2-fold (light blue bars) and 5-fold (dark blue bars) molar excess of NHS-ester.

On the other hand, measurements of blockade efficacy for antibodies that recognize the growth factors were considerably affected by the labeling method (Fig. 8 and see Supplementary Fig. S4). Blockade efficacies derived from interactions of these antibodies with NHS-ester-labeled growth factors were generally lower than those derived from interactions with their CBT-labeled counterparts. These results suggest that the heterogeneous NHS-ester labeling may alter to different extent epitopes that are necessary for antibody recognition. We further tested this by comparing the capacity of BRET assays utilizing three batches of CBT or NHS-ester-labeled VEGF_165_a to provide reliable and reproducible blockade measurements for two antibodies MAB293 (Fig. 8a) and Bevacizumab (Fig. 8b). Blockade efficacies derived from assays utilizing three batches of CBT-labeled VEGF_165_a were remarkably reproducible (K_I_’s: 0.05 ± 0.004 nM and 0.2 ± 0.012 nM for MAB293 and Avastin, respectively), exhibiting less than 10% batch-to -batch variability (Fig. 8c). In contrast, a similar analysis utilizing three batches of VEGF_165_a labeled with 2 or 5-fold molar excess of DY605-NHS-ester revealed significantly lower blockade efficacies for MAB293 (K_I_’s: 0.54 ± 0.3 nM and 1.3 ± 0.3 nM, respectively) and Bevacizumab (K_I_’s: 1 ± 0.5 nM and 1.4 ± 0.3 nM, respectively) as well as high batch-to-batch variability (50% and 20%, respectively) (Fig. 8c). Taken together, this comparative analysis further demonstrates the advantages of the CBT-labeling method for robust, reliable and quantitative protein ligand-binding assays.

## Discussion

The capacity of fluorescently-labeled protein ligands to facilitate detection and quantitation of binding events is widely recognized. Accordingly, simple methods for reproducible, homogenous and efficient protein labeling that minimally interfere with protein function are highly desired. Herein, we demonstrated the capabilities of a single-step method, integrating HaloTag protein purification and CBT condensation, to reproducibly generate homogenous populations of fluorescently-labeled growth factors. LC-MS/MS analysis revealed that these growth factors were quantitatively labeled with a single fluorophore on their N-terminal cysteine. Measurements of ligand binding and transcriptional activation indicated that these labeled growth factors retained biological activities that were equivalent to those of their unlabeled counterparts. This demonstrated the minimally disruptive nature of the site-specific CBT-labeling approach. Notably, this labeling method is not restricted to growth factors and could be applied to other proteins able to tolerate modification at their N-terminus.

We demonstrated the benefits of this reproducible, homogeneous and minimally perturbing labeling method through the development of a novel assay quantifying antibody blockade. The assay exploits the exquisite sensitivity of BRET for detecting molecular proximity, which enables measurements of interaction between a NanoLuc-tagged receptor and its fluorescently labeled ligand. This provides the means for quantifying an antibody’s capacity to block receptor-ligand interaction through a decrease in BRET. The inherent distance constraints of BRET provide the specificity required for monitoring antibody blockade on the surface of living cells, using full-length receptors in their native conformation. Moreover, binding properties of the corresponding native protein ligands may be suitably emulated by labeled ligands generated using our CBT-labeling method. Hence, this assay configuration can reliably represent endogenous receptors and ligands that the antibodies are intended to recognize and interactions they meant to disrupt. Using several therapeutic and research antibodies, we validated the capacity of this assay to reliably quantify blockade efficacies for antibodies that target the receptors or their ligands. We expect that this simple assay for quantifying biophysical antibody blockade in a cellular context, will complement the collection of assays already being used during development of therapeutic antibodies. These include functional assays reporting on a distal event that can be affected by multiple pathways and biochemical antibody binding assays that do not report of blockade efficacy.

Finally, we showed that the capacity to generate homogenously labeled-growth factors with well-characterized binding properties supported assay robustness and reproducibility. Furthermore, the ability to generate these labeled-growth factors without compromising their binding properties contributed to the quality and reliability of the assay, particularly when assay outcome relied on direct binding of antibodies to the labeled growth factors. Taken together, this simple method for quantitative labeling of protein ligands with a single fluorophore or other chemical entities without compromising their function, has the potential to promote more accurate and robust ligand binding assays.

## Methods

See Supplemental Information for detailed information about material and methods related to chemical synthesis of CBT conjugate, generation of DNA constructs and cell culture.

### Materials

Recombinant human EGF and PDGF-BB were from R&D Systems and recombinant human VEGF_165_a was from Life technologies. Research antibody targeting EGF was from Cell Signaling and research antibodies targeting PDGF-BB, PDGFRβ and VEGF_165_a were from R&D systems. The therapeutic antibodies Cetuximab, Panitumumab, Bevacizumab and Trastuzumab were from Myoderm and DY605-NHS-ester was obtained from DYOMICS GmbH.

### Synthesis and purification of labeled and unlabeled growth factors

The growth factors were transiently expressed as secreted HaloTag fusion proteins in 1 L of HEK293T cells and purified using the HaloTag mammalian protein detection and purification system (Promega) according to manufacturer recommendations. Briefly, following 72 h of expression, the media containing the secreted fusion protein was collected and incubated with 1.8 mL of pre-equilibrated HaloLink beads. Covalent binding to the beads was carried out for 16–20 h at 4 °C with constant end-over-end rotation. Following three washes of the beads, 10 min each, the growth factors were released from the beads by proteolytic cleavage using 700 units of HaloTEV in the presence of 100 µM TCEP (Thermo Scientific). The proteolytic reaction was conducted at 4 °C for 16 h with constant mixing. Generation of CBT-labeled growth factors was performed according to our published protocol^17^, where HaloTEV proteolytic release was carried out in the presence of 4-fold molar excess DY605-PEG-CBT over the expressed fusion protein. For the generation of NHS-ester-labeled growth factors, purified proteins were dialyzed for 16–18 h (100 mM sodium bicarbonate pH 8.6 and 200 mM NaCl) prior to 15 min labeling at 4 °C in the presence of 2 and 5-fold molar excess of DY605-NHS-ester over protein^6^. To determine the amounts (moles) of DY605-PEG-CBT or DY605-NHS-ester required for labeling, the moles of expressed HaloTag fusions were determined as previously described^13^. Purified labeled and unlabeled growth factors were dialyzed for 16–20 h (50 mM HEPES and 150 mM NaCl) to remove the unconjugated dye and TCEP. The purified proteins were stored in the presence of 2.5 mg mL^−1^ bovine serum albumin (Millipore) at −80 °C.

### Liquid chromatography–tandem mass spectrometry (LC–MS/MS) analysis

Equal amounts of labeled and unlabeled growth factors purified in the same manner were resolved by SDS-PAGE. Gel slices were washed and reduced with 25 mM TCEP in 50 mM Tris, pH 8.0 at 56 °C for 20 min. Following reduction, proteins were in-gel digested with either trypsin, elastase or LysC/GluC (Promega) (at 12 µg/mL protease in 50 mM Tris, pH 8.0 supplemented with 0.01% Protease Max (Promega) for 2 h at 37 °C with constant mixing. Reactions were quenched with 0.5% TFA and subjected to LC-MS/MS analysis on a Q Exactive Hybrid Quadrupole-Orbitrap Mass Spectrometer (Thermo Scientific) operating with a MS resolution of 70,000. MS-product component of the Protein Prospector software (University of California) was used to calculate the mono-isotopic masses of the modified and unmodified peptides at different charge states, (Z = 2–5 were considered). Extracted Ion Chromatograms were generated using Xcalibur (Thermo Scientific). Peptides containing modified residues displayed increased mass of 1434.42 Da or 973.23 Da for a DY606-PEG-CBT or DY605-NHS-ester modification, respectively. For each modified residue, the degree of labeling was estimated by assessing the fraction that remained unmodified and subtracting it from a theoretical maximum of 100% labeling. To this end, equal amounts of labeled and unlabeled samples were compared for the relative abundance of unmodified peptides encompassing those residues (i.e. N-terminal cysteine or lysine shown to be modified). Relative abundances were derived from the ratio between the integrated peak areas in the Extracted Ion Chromatograms of those unmodified peptides, where the integrated peak area from unlabeled samples represented 100% peptide abundance. The relative abundances of those unmodified peptides in the labeled samples corresponds to the fractions of N-terminal cysteines and lysines that were not modified (see Supplementary Tables S1 and S2).

### NFAT and SRE reporter gene assays

HEK293 cells stably expressing a firefly luciferase reporter gene (NFAT-RE-Luc2P or SRE-Re-Luc2P from Promega) and transiently expressing a relevant NanoLuc:RTK fusion were used to monitor NFAT or SRE induced transcription following stimulation with labeled and unlabeled growth factors. 24 h post transfection, cells were collected re-suspended in serum free media, seeded into white 96 well plates at density of 4 × 10^4^ cells/well and incubated for 2 h at 37 °C, 5% CO_2_. Cells were then stimulated in triplicates for 6 h at 37 °C, 5% CO_2_ with increasing concentrations of labeled and unlabeled growth factors. 10-fold serial dilutions of labeled and unlabeled growth factors were prepared in OptiMEM + 1% BSA. Expression of reporter genes was measured using ONE-Glo luciferase reagent (Promega) according to manufacturer recommendations. Apparent potencies (EC50) were determined using curve fits in GraphPad Prism with the equation:

$${\rm{Y}}=\frac{{\rm{Bottom}}+({\rm{Top}}-{\rm{Bottom}})}{{1+10}^{(({\rm{LogEC50}}-{\rm{X}})\times \text{HillSlope})}}$$

### Measuring ligand binding by BRET

HEK293T cells were transfected with DNA constructs encoding a NanoLuc:RTK fusion (diluted 1:100 with carrier plasmid). 24 h post transfection, cells were collected, re-suspended in OptiMEM without phenol red and seeded into white non-binding 96 well plates at a density of 2 × 10^4^ cell/well. To determine binding affinity of labeled growth factors for their cognate RTKs, cells were treated with increasing concentration of labeled growth factors in the presence and absence of excess (0.5 µM) unlabeled equivalents. 10X serial dilutions of labeled growth factors were prepared in OptiMEM + 1% BSA. Following 1.5 h binding at room temperature, BRET was measured by the addition of NanoBRET NanoGlo substrate at a final 1:500 dilution. Filtered luminescence was measured on a Varioskan luminometer (Thermo Scientific) equipped with a 450-nm band pass filter (donor) and a 610-nm long pass filter (acceptor), using 0.5 sec integration time. BRET ratios were determined by dividing the 610 nm signals by the 450 nm signals. Background-corrected BRET ratios were determined by subtracting the BRET ratios of samples treated with excess competing unlabeled growth factors from the BRET ratios in the absence of competing unlabeled growth factors. Apparent affinities (EC50) were determined using curve fits in GraphPad Prism with the equation:

$${\rm{Y}}=\frac{{\rm{Bottom}}+({\rm{Top}}-{\rm{Bottom}})}{{1+10}^{(({\rm{LogEC50}}-{\rm{X}})\times \text{HillSlope})}}$$

Normalized data were generated by assigning 100% to the theoretical maximum of the curve fit and 0% for the theoretical minimum value of the curve fit. Binding constants (K_D_) were determined using curve fits in GraphPad Prism with the equation:

$${\rm{Y}}=\frac{{\rm{Bmax}}\,\times \,{\rm{X}}}{{\rm{KD}}+{\rm{X}}\,}$$

### Measuring efficacy of antibody blockade by BRET

HEK293T cells were transfected with DNA encoding a NanoLuc:RTK fusion, collected and re-plated as described above. For blockade experiments, cells were simultaneously incubated with fixed EC80 concentrations of CBT-labeled growth factors and increasing concentrations of antibodies. Following 1.5 h binding at room temperature, BRET measurements were performed as described above. Results for competitive displacement were graphed with GraphPad Prism with the equation:

$${\rm{Y}}=\frac{{\rm{Bottom}}+({\rm{Top}}-{\rm{Bottom}})}{{\rm{1}}+{{\rm{10}}}^{(({\rm{LogIC50}}-{\rm{X}})\times \mathrm{HillSlope})}}$$

Apparent blockade constants were calculated for each antibody from the observed IC50 values according to the Cheng-Prusoff equation^32^, where L is the concertation of the labeled growth factor and K_D_ is the binding affinity of the labeled growth factor calculated from the saturation binding experiments described above.

$${{\rm{K}}}_{{\rm{I}}}=\frac{{\rm{IC}}50}{1+\frac{[{\rm{L}}]}{{{\rm{K}}}_{{\rm{D}}}}}$$

## Supplementary information

Utilizing a Simple Method for Stoichiometric Protein Labeling to Quantify Antibody Blockade
