# Supplementary material for: Utilizing a Simple Method for Stoichiometric Protein Labeling to Quantify Antibody Blockade
Source: Sci Rep. 2019 May 7;9:7046. doi: 10.1038/s41598-019-43469-z (PMC6504924; doi:10.1038/s41598-019-43469-z)
Supplement: Supplementary file 1 — Utilizing a Simple Method for Stoichiometric Protein Labeling to Quantify Antibody Blockade [file 41598_2019_43469_MOESM1_ESM.pdf]

Supplementary Information

Utilizing a Simple Method for Stoichiometric Protein Labeling to Quantify Antibody Blockade

Rachel Friedman Ohana[1*], Robin Hurst[1], Mike Rosenblatt[1], Sergiy Levin[2], Thomas Machleidt[1], Thomas A. Kirkland[2], Lance P. Encell[1], Matthew B. Robers[1], and Keith V. Wood[1]

[1]Promega Corporation, 2800 Woods Hollow Rd, Madison, WI 53711
[2]Promega Biosciences LLC, 277 Granada Dr, San Luis Obispo, CA 93401

Supplementary information is divided into two parts (excluding Supplementary References): Supplementary Methods and Supplementary Figures and Tables. The later is listed according to the main text and the main figures to which they are related.

Content:

Supplementary Methods:
1. Synthesis of DY605-PEG-CBT conjugate
2. Constructs for producing labeled and unlabeled growth factors
3. Constructs for genetic fusions of NanoLuc with receptor tyrosine kinases
4. Cell culture and transfections
5. Dimerization assay

Supplementary Figures and Tables
1. Figure 1. Dimerization state of labeled growth factors.
2. Figure 2. Peptide coverage by LC-MS/MS analysis of growth factors digested with multiple proteases.
3. Table 1. LC-MS/MS profile of unmodified peptides from proteolytic digestion of unlabeled and CBT-labeled growth factors.
4. Table 2. LC-MS/MS profile of unmodified peptides from proteolytic digestion of unlabeled and NHS-ester-labeled growth factors.
5. Figure 3. Binding constants of labeled and unlabeled growth factors for their cognate receptors.
6. Figure 4. Influence of labeling method on quantitative assessment of antibody blockades.
7. Figure 5. Uncropped images for Figure 2 and Supplementary Figure 1.

Supplementary Methods

1. Synthesis of CBT-PEG-DY605 conjugate

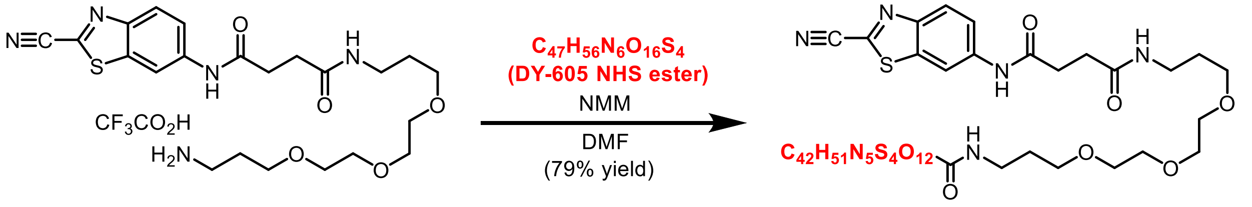

To a solution of **S01**[1](2.7 mg, 4.6 µmol) in DMF (2 mL), **Dyomics DY-605 NHS ester** (5.0 mg, 4.6 µmol) was added, followed by a drop of N-Methylmorpholine (NMM), and the resulting solution was stirred at 22 °C for 120 minutes, at which point HPLC analysis indicated consumption of the starting material. The reaction mixture was directly loaded onto C18 column and purified by preparative HPLC (C_{18}, 20 → 60% MeCN/H_2O, 0.05% TFA). Solvent was removed by lyophilization to provide 5.3 mg (80 % yield) of **DY605-PEG-CBT (S02)**, as deep purple solid. MS (ESI+) calc'd for $C_{65}H_{83}N_{10}O_{18}S_5^+$ $[M+H]^+$ 1451.45, found 1451.05; HPLC: 99% @ 600 nm.

2. Constructs for producing labeled and unlabeled growth factors

cDNAs for EGF, PDGF-B and $VEGF_{165}$a were synthetically synthesized by Gene Dynamics LLC and subcloned into a modified pFN21 HaloTag CMV Flexi vector carrying an IL6 secretion signal. The linker sequence separating the two fusion partners was replaced with a linker encoding EPTTEDLYFQCDN.

3. Constructs for genetic fusions of NanoLuc with receptor tyrosine kinases

N-terminal NanoLuc fusions of EGFR, PDGFRβ and VEGFR2 were generated by cloning the protein coding regions without their native signal sequence into pNKF1-secN using the Flexi Vector cloning system (Promega). The ORFs were obtained from Kazusa DNA Research Institute.

4. Cell culture and transfections

HEK293T and HEK293 cells were grown in DMEM medium (Sigma) supplemented with 10% FBS (Hyclone) at 37 °C, 5% CO_2, except HEK293 stably expressing a reporter gene were grown in the presence of 50 µg mL^{-1} of Hygromycin B (Fisher Scientific). All transient transfections were performed as previously described[2] with 0.8 µg mL^{-1} DNA constructs using PEI transfection reagent, at a PEI (Polysciences, Inc.) to cDNA ratio of 3:1. To reduce expression levels, DNA constructs encoding NLuc

fusions were diluted 1:100 with a promoterless carrier DNA plasmid (pCI-neo; Promega) to generate a final total DNA concentration of 0.8 µg mL^{-1}.

5. Dimerization assay

Purified labeled growth factors were analyzed on SDS-PAGE in the presence or absence of 100 mM DTT and then scanned on a Typhoon FLA9500 fluorescent imager (GE Healthcare), (Excitation = 600 nm; Emission = 624 nm).

Supplementary Figures and Tables

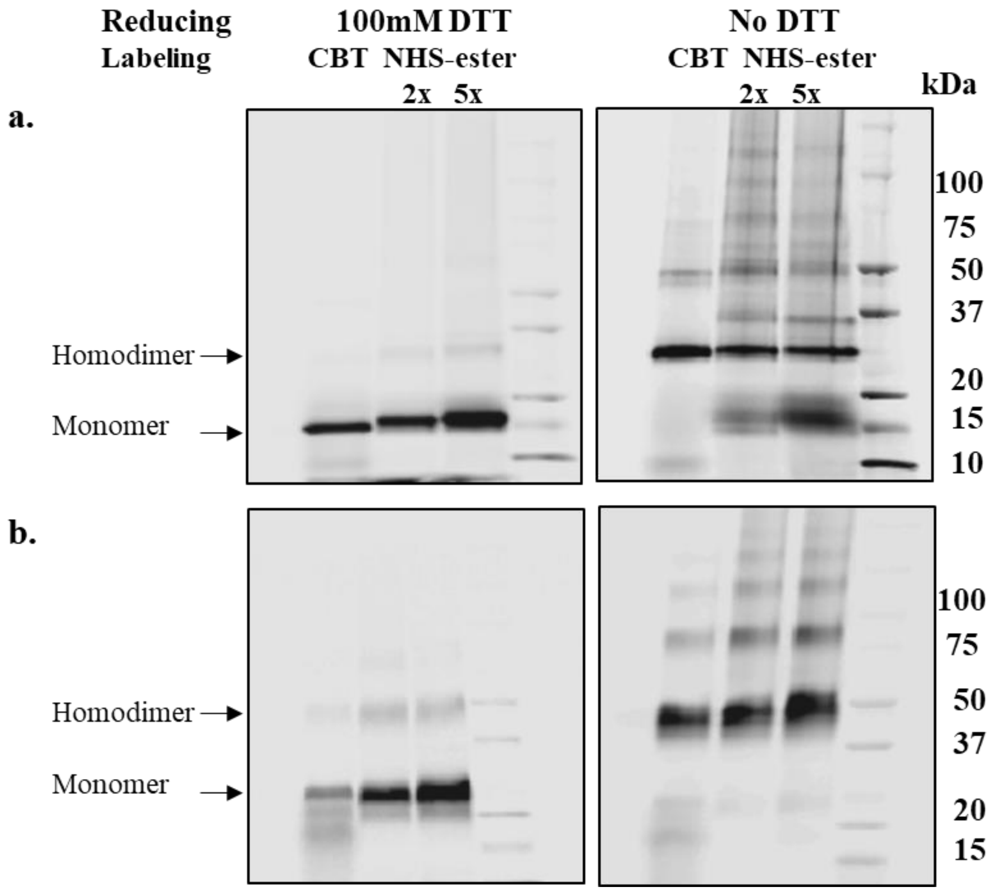

Figure 1. Dimerization state of labeled growth factors. Fluorescent SDS-PAGE analysis of (**a**) PDGFB and (**b**) $VEG_{165}a$. Growth factors labeled by CBT or 2 and 5-fold molar excess of NHS-ester were resolved on SDS-PAGE in the presence or absence of 100 mM DTT and scanned on a Typhoon FLA9500 (GE Healthcare) using the Cy3 setting. Uncropped images are shown in Supplementary Fig. 5 on line.

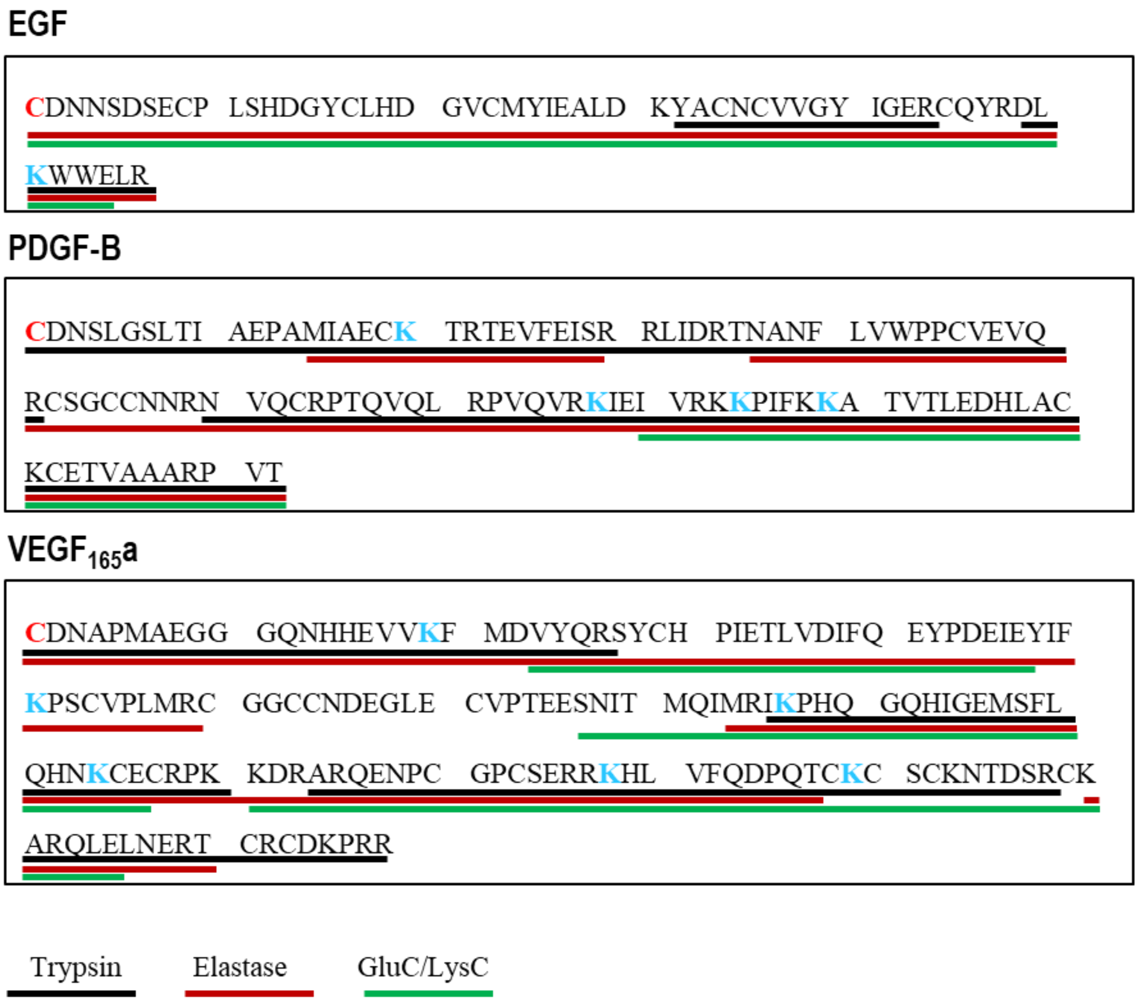

Figure 2. Peptide coverage by LC-MS/MS analysis of growth factors digested with multiple proteases. Peptide coverage achieved from in-gel digestion with Trypsin, Elastase and GluC/LysC. The N-terminal cysteine is marked in red and modified lysine residues are marked in light blue. Protein identities were confirmed by searching the MS/MS spectra with Mascot search engine (Matrix Sciences Inc.) against the human database (SwissPort). The highest scoring hits were the relevant growth factor sequences.

Table 1. LC-MS/MS profile of unmodified peptides from proteolytic digestion of unlabeled and CBT-labeled growth factors.

Protease	Peptide Sequence	Residue	Sample	Peak Area	%	% labeled
**EGF**						
LysC/GluC	**C**DNNSDSECPLSHD	Cys1	unlabeled	9.8E+07	100	
			CBT	4.0E+06	4.1	95.9
**PDGF-B**						
Trypsin	**C**DNSLGSLTIAEPAMIAECK	Cys1	unlabeled	3.5E+09	100	
			CBT	5.2E+07	1.5	98.5
**VEGF$_{165}$a**						
Trypsin	**C**DNAPMAEGGGQNHHEVVK	Cys1	unlabeled	1.5E+08	100	
			CBT	1.9E+06	1.2	98.8

Labeling efficiencies were estimated by assessing the fractions of N-terminal cysteines that remained unmodified and subtracting them from a theoretical maximum of 100% labeling. To this end, equal amounts of labeled and unlabeled samples were compared for the relative abundance of unmodified peptides encompassing the N-terminal cysteines. Relative abundances were derived from the ratio between the integrated peak areas of those unmodified peptides, where the integrated peak area from unlabeled samples represented 100% peptide abundance. The relative abundances of those unmodified peptides in the labeled samples corresponds to the fractions of N-terminal cysteines that were not modified.

Table 2. LC-MS/MS profile of unmodified peptides from proteolytic digestion of unlabeled and NHS-ester-labeled growth factors.

Protease	Peptide Sequence	Residue	Sample	Peak Area	%	% labeled
**EGF**						
LysC/GluC	**C**DNNSDSECPLSHD	N-term	unlabeled	1.4E+09	100	
			2 ×-NHS	6.3E+08	44.3	55.7
			5 ×-NHS	1.7E+08	12.1	87.9
Elastase	QYRDL**K**WWEL	Lys 51	unlabeled	6.9E+12	100	
			2 ×-NHS	3.4E+12	48.8	51.2
			5 ×-NHS	9.0E+10	1.3	98.7
**PDGF-B**						
Elastase	**K**TRTEVFEI	Lys 20	unlabeled	1.1E+11	100	
			2 ×-NHS	1.0E+11	96.3	3.7
			5 ×-NHS	6.2E+10	57.6	42.4
Elastase	R**K**IEIVR	Lys 77	unlabeled	7.1E+08	100	
			2×-NHS	2.1E+08	29.6	70.4
			5×-NHS	1.9E+08	26.8	73.2
Trypsin	K**K**PIFKK	Lys 84	unlabeled	3.4E+09	100	
			2 ×-NHS	1.1E+09	31.5	68.5
			5 ×-NHS	1.0E+09	29.4	70.6
Trypsin	**K**ATVTLEDHLA**C**K	Lys 89	unlabeled	4.6E+07	100	
			2 ×-NHS	2.1E+06	4.5	95.5
			5 ×-NHS	1.9E+06	4.1	95.9
**VEGF$_{165}$a**						
Trypsin	CDNAPMAEGGGQNHHEVV**K**	Lys 19	unlabeled	1.4E+09		
			2 ×-NHS	1.4E+09	98.6	1.4
			5 ×-NHS	9.6E+08	68.8	31.2
Elastase	EYIF**K**PS	Lys 51	unlabeled	7.1E+09		
			2 ×-NHS	3.2E+09	45.1	54.9
			5 ×-NHS	9.5E+08	13.4	86.6
Elastase	MRI**K**PHQGQH	Lys 87	unlabeled	7.2E+08		
			2 ×-NHS	4.2E+08	58.2	41.8
			5 ×-NHS	7.7E+07	10.8	89.2
LysC/GluC	MSFLQHN**K**	Lys 104	unlabeled	1.1E+09		
			2 ×-NHS	2.5E+07	2.3	97.7
			5 ×-NHS	2.4E+02	0.0	100
Elastase	SERR**K**HLFV	Lys 128	unlabeled	5.6E+09		
			2 ×-NHS	4.8E+06	0.1	99.9
			5 ×-NHS	2.2E+07	0.4	99.6
LysC/GluC	HLFVQDPQTC**K**	Lys 139	unlabeled	3.8E+07		
			2 ×-NHS	6.0E+06	15.6	84.4
			5 ×-NHS	5.5E+06	14.4	85.6

Labeling efficiencies were estimated by assessing the factions of lysine residues that were not modified and subtracting them from a theoretical maximum of 100% labeling. To this end, equal amounts of labeled and unlabeled samples were compared for the relative abundance of unmodified peptides encompassing modified lysines (bolded). Relative abundances were derived from the ratio between the integrated peak areas of those unmodified peptides, where the integrated peak area from unlabeled samples represented 100% peptide abundance. The relative abundances of those unmodified peptides in the labeled samples corresponds to the fractions of lysine residues that were not modified.

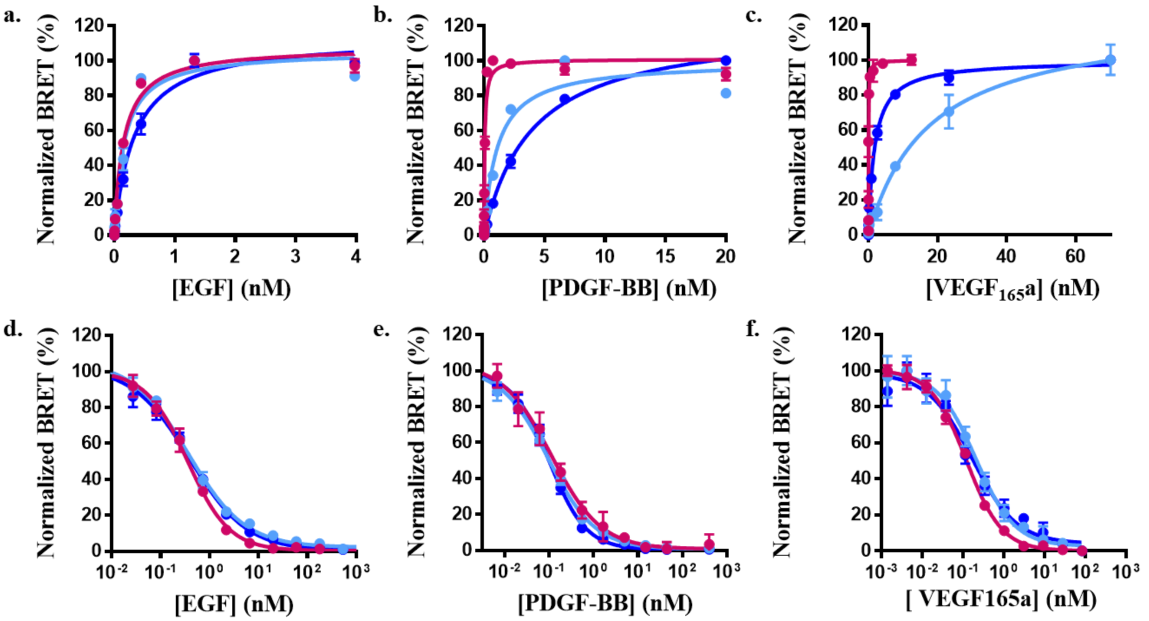

g.

Labeling	EGF	PDGF-BB	VEGF$_{165}$a
CBT labeled ligand			
K_D (pM)	160	62	49
EC80 (pM)	200	100	76
Unlabeled ligand			
IC50 (pM)	350	110	120
K_I (pM)	150	44	47

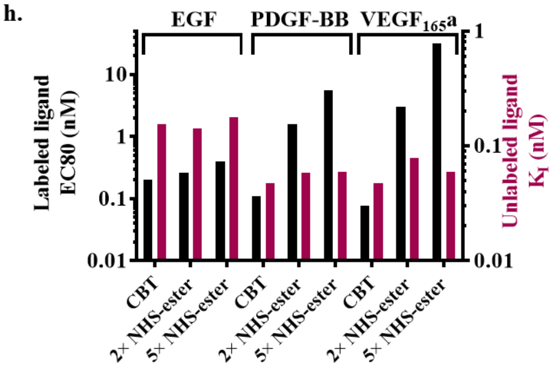

Figure 3. Binding constants of unlabeled and labeled growth factors for their cognate receptors. Saturation binding of increasing concentrations of (**a**) EGF, (**b**) PDGF-BB and (**c**) VEGF$_{165}$a labeled by CBT (•) or 2-fold (•) and 5-fold (•) molar excess of NHS-ester to their cognate receptors that are genetically fused to NanoLuc. Data expressed as normalized corrected BRET ratios was used to derive binding constants (K_D) for labeled growth factors. Competitive displacements of fixed EC80 concentrations of labeled (**d**) EGF, (**e**) PDGF-BB and (**f**) VEGF$_{165}$a by increasing concentrations of unlabeled equivalents. Data expressed as normalized BRET ratios was used to derive IC50 values and to calculate binding constants (K_I) for unlabeled growth factors according to the Cheng-Prusoff equation[3]. (**g**) Binding constants of unlabeled and CBT-labeled growth factors. (**h**) Summary of EC80 concentrations of labeled growth factors used in displacement experiments (black bars) to derive IC50's and calculate K_I values for unmodified compounds according to Cheng-Prusoff equation (red bars).

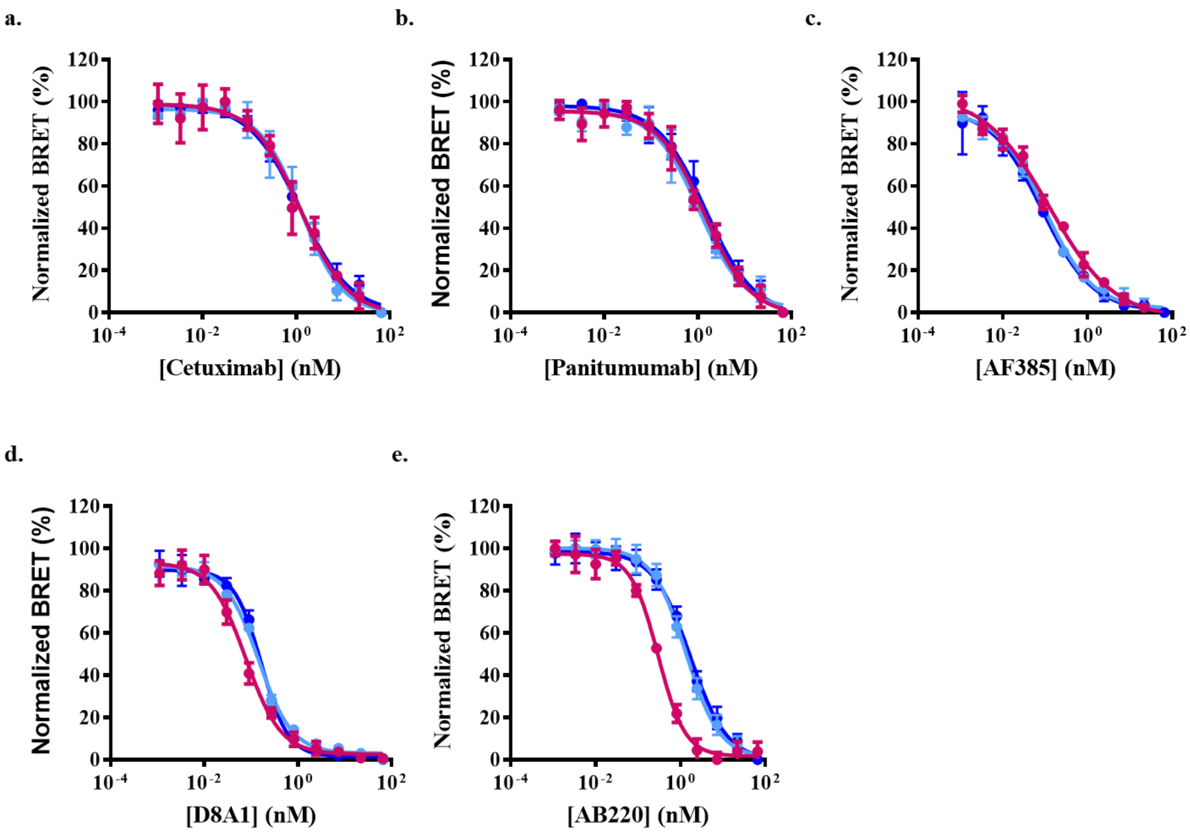

Figure 4. Influence of labeling method on quantitative assessment of antibody blockades. BRET-based assay quantifying blockade for antibodies recognizing the receptors (**a**) Cetuximab, (**b**) Panitumumab and (**c**) AF385 as well as antibodies recognizing the growth factors (**d**) D8A1 and (**e**) AB220. Cells transiently expressing the relevant NanoLuc:RTK fusion were treated simultaneously with fixed concentrations (EC80) of growth factors labeled by CBT (•) or 2-fold (•) and 5-fold (•) molar excess of NHS-ester and increasing concentrations of antibodies. Data is expressed as normalized BRET ratios (n=4).

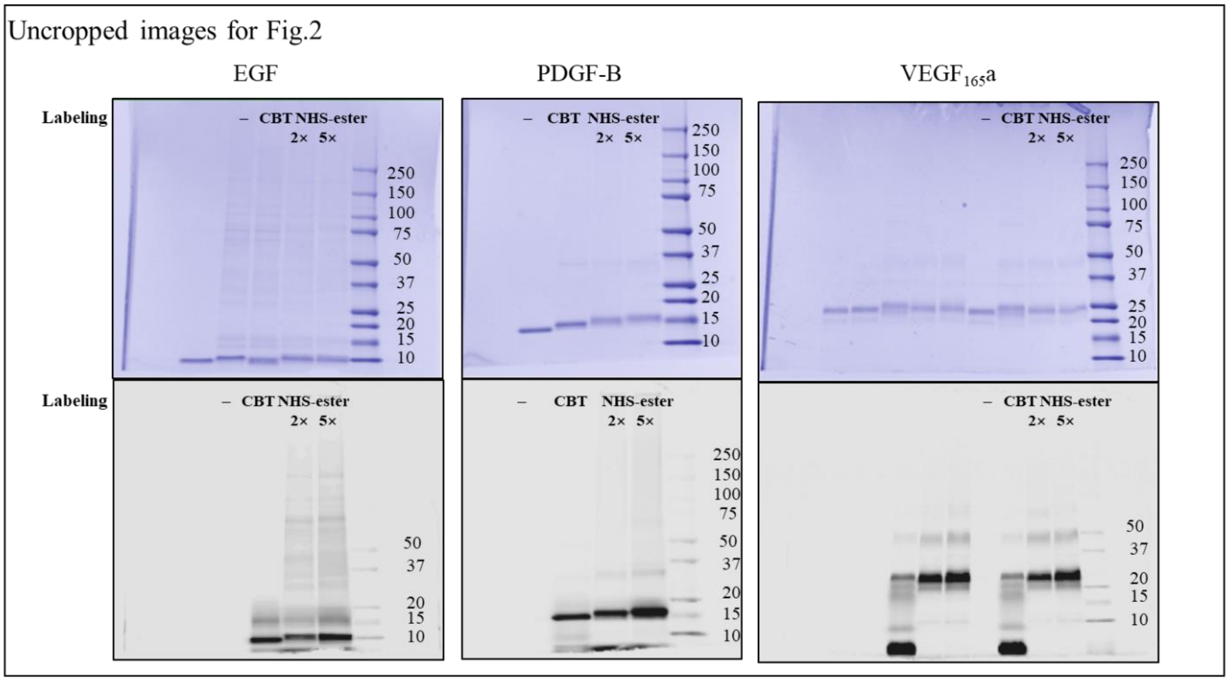

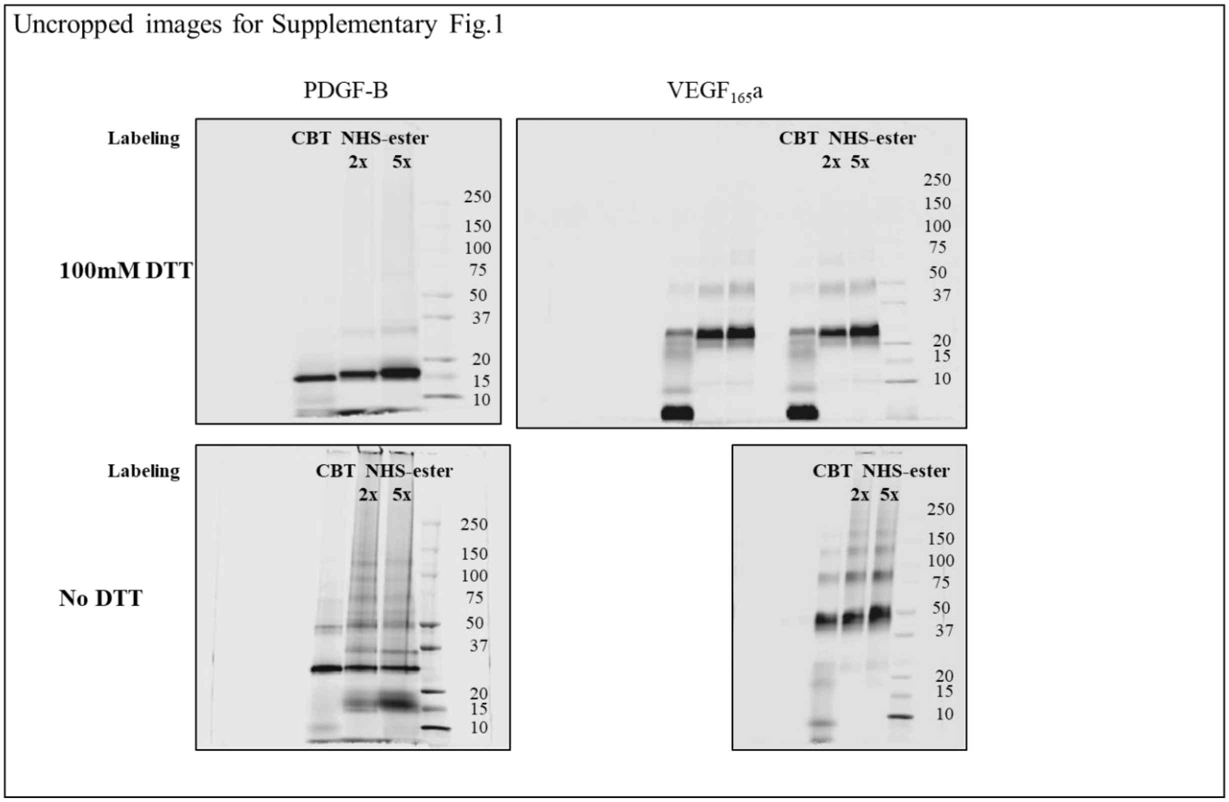

Figure 5. Uncropped images for Figure 2 and Supplementary Figure 1. Relevant lanes are annotated.

Refferences

1. Friedman Ohana, R. *et al.* Deciphering the cellular targets of bioactive compounds using a chloroalkane capture tag. *ACS Chem Biol* **10**, 2316-2324, doi:10.1021/acschembio.5b00351 (2015).
2. Ohana, R. F. *et al.* HaloTag-based purification of functional human kinases from mammalian cells. *Protein expression and purification* **76**, 154-164, doi:10.1016/j.pep.2010.11.014 (2011).
3. Cheng, Y. & Prusoff, W. H. Relationship between the inhibition constant (K1) and the concentration of inhibitor which causes 50 per cent inhibition (I50) of an enzymatic reaction. *Biochem Pharmacol* **22**, 3099-3108 (1973).
